# Gentamicin Sulfate Grafted Magnetic GO Nanohybrids with Excellent Antibacterial Properties and Recyclability

**DOI:** 10.3390/nano13081416

**Published:** 2023-04-20

**Authors:** Xing Wu, Jingya Zhou, Zeng Liu, Wei Shao

**Affiliations:** Jiangsu Co-Innovation Center of Efficient Processing and Utilization of Forest Resources, College of Chemical Engineering, Nanjing Forestry University, Nanjing 210037, China

**Keywords:** magnetic, graphene oxide, gentamicin sulfate, antibacterial, recyclable activity

## Abstract

In this study, magnetic graphene oxide (MGO) nanohybrids were first prepared by loading Fe_3_O_4_ NPs onto graphene oxide (GO). Then, GS-MGO nanohybrids were prepared by grafting gentamicin sulfate (GS) onto MGO directly using a simple amidation reaction. The prepared GS-MGO had the same magnetism as MGO. They exhibited excellent antibacterial ability against Gram-negative bacteria and Gram-positive bacteria. The GS-MGO had excellent antibacterial performance against *Escherichia coli* (*E. coli*), *Staphylococcus aureus* (*S. aureus*), and *Listeria monocytogenes* (*L. monocytogenes*). When the addition concentration of GS-MGO was 1.25 mg/mL, the calculated bacteriostatic ratios against *E. coli* and *S. aureus* achieved 89.8% and 100%, respectively. For *L. monocytogenes*, only 0.05 mg/mL of GS-MGO had an antibacterial ratio as high as 99%. In addition, the prepared GS-MGO nanohybrids also exhibited excellent non-leaching activity with good recycling antibacterial ability. After eight times antibacterial tests, GS-MGO nanohybrids still exhibited an excellent inhibition effect on *E. coli*, *S. aureus,* and *L. monocytogenes*. Therefore, as a non-leaching antibacterial agent, the fabricated GS-MGO nanohybrid had dramatic antibacterial properties and also showed great recycling ability. Thus, it displayed great potential in the design of novel recycling antibacterial agents with non-leaching activity.

## 1. Introduction

Microbial contamination has a huge influence on food and human health and also has an enormous technical impact on the market economy and medical standards [1,2,3]. There is no doubt that the development of novel antibacterial agents has become a necessary means to ensure health [4,5]. In the past decade, various antibiotics have been developed using methods that mainly rely on the release of antibiotics to achieve the purpose of inhibiting microbial reproduction [6]. However, the overuse of antibiotics has increased the resistance of microorganisms and enhanced the threat of bacteria to the human body [7,8,9]. With the rapid development of nanotechnology in materials, non-leaching antibacterial agents have gradually appeared in public view.

Graphene has a perfect hybrid structure and large conjugated system, which gives it strong electron transport ability [10,11]. However, its strong hydrophobicity limits its practical applications. In comparison, graphene oxide (GO) has a large number of functional groups, such as hydroxyl and carboxyl groups. Additionally, GO has a huge surface area, thermal stability, layered structure, good chemical stability, and antibacterial performance [12]. In order to strengthen the antibacterial performance of GO, various works were carried out. Copper oxide nanoparticles were loaded onto GO in order to improve the antibacterial activity, and the prepared GO-Cu NPs displayed excellent antibacterial activity at extremely low concentrations, which could be applied as an antibacterial agent [13]. A zinc oxide-reduced GO nanocomposite was synthesized aiming to provide great antibacterial behavior. A synergy in bacterial activity between ZnO and reduced GO was demonstrated toward waterborne Gram-negative and Gram-positive bacteria [14]. Silver nanoparticles doped GO were fabricated using glucose as an eco-friendly reducing agent using a one-pot method and exhibited outstanding antibacterial performance, which affirmed their future use not only as an efficient antibacterial agent but also potentially as a novel anti-virus treatment [15].

There is no doubt that antibiotics display good antibacterial abilities. Gentamicin sulfate (GS) is an aminoglycoside antibiotic that acts on ribosomes in bacteria and inhibits bacterial protein synthesis until the integrity of bacterial cell membranes is destroyed [16]. Therefore, GS has frequently been used to enhance the antibacterial ability of different materials. Gentamicin-grafted chitosan film was produced, and the GS grafting treatment significantly improved the antibacterial properties of the chitosan film [17]. GS was loaded into silk fibroin-mediated porous chitosan films, and the prepared films exhibited broad-spectrum antibacterial activity, appropriate biocompatibility, and desirable polar interaction with the GS for controlled drug release applications [18].

The excessive and improper applications of antibiotics have resulted in the appearance of drug-resistant superbugs in recent years. Therefore, the use of GS has been minimized in clinical applications due to being a kind of antibiotic. The development of antibacterial agents without any leaching effect is of great interest. In this work, our objective was to develop excellent antibacterial, stable, and recyclable GO-based nanomaterials without any GS leaching. The magnetic GO (MGO) was first prepared by loading with Fe_3_O_4_ NPs to endow it with magnetism. Then, the GS-MGO was prepared by grafting GS onto MGO through the amidation reaction. The fabricated GS-MGO demonstrated superior antibacterial activities against *E. coli*, *S. aureus,* and *Listeria monocytogenes* (*L. monocytogenes*) without any leaching effect. Additionally, the dramatic reusability of GS-MGO was confirmed by eight-times antibacterial applications towards Gram-positive and Gram-negative bacteria. They displayed great potential applications in clinical environmental applications and food packaging fields.

## 2. Materials and Methods

### 2.1. Materials

GO was purchased from XFNANO Materials Tech Co., Ltd. (Nanjing, China). NH_4_Fe(SO_4_)_2_·12H_2_O, FeSO_4_·7H_2_O, *N*-Hydroxysuccinimide (NHS), and NH_3_·H_2_O (NH_3_: 25.0~28.0%, AR) were purchased from Sinopharm Chemical Reagent Co., Ltd. (Shanghai, China). *N*-(3-Dimethylaminopropyl)-*N′*-ethylcarbodiimide hydrochloride (EDC) was purchased from Macklin Biochemical Co., Ltd. (Shanghai, China). MES monohydrate (MES) and GS were purchased from Aladdin Biochemical Technology Co., Ltd. (Shanghai, China).

### 2.2. GS-MGO Preparation

**Figure 1:**
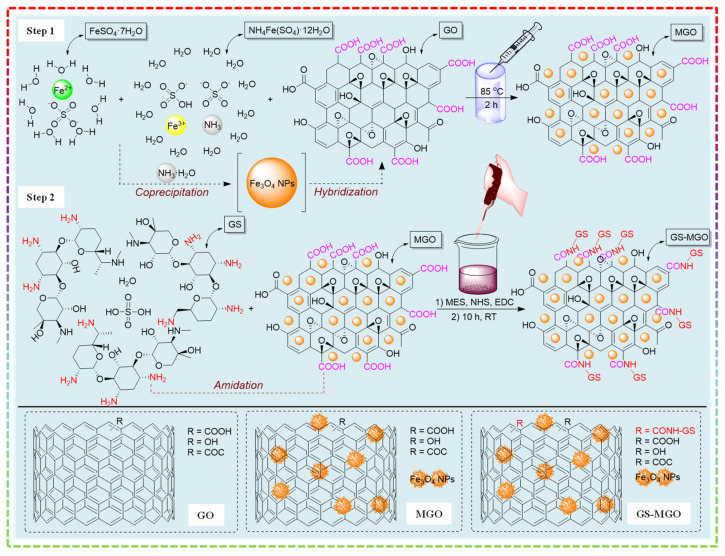
Schematic diagram of the preparation process of the GS−MGO nanohybrids.

The preparation process of GS-MGO is shown in Figure 1. Firstly, FeSO_4_·7H_2_O (0.14 g) and NH_4_Fe(SO_4_)_2_·12H_2_O (0.48 g) were dissolved into 10 mL deionized (DI) water to obtain 0.05 M FeSO_4_ and 0.1 M NH_4_Fe(SO_4_)_2_ solution, respectively. GO suspension was obtained by adding GO (0.2 g) into 100 mL DI water with an Ultrasonicator (Ymnl-1000Y, Nanjing, China) for 120 min. Then, 10 mL 0.05 M FeSO_4_, 10 mL 0.1 M NH_4_Fe(SO_4_)_2_, and 15 mL NH_3_·H_2_O were added into the GO suspension by stirring at 85 °C for 2 h. Then the mixed solution was centrifugated at 10,000 rpm for 10 min, followed by washing with DI water three times. The MGO was gained from freeze-drying at −70 °C for 24 h.

A total of 0.1 g EDC and 0.2 g NHS were taken to 0.1 M MES buffer solution (pH = 5.5) and stirred for 10 min. Then, 0.1 g GS and 0.1 g MGO were added and stirred for 10 h, followed by centrifugation at 10,000 rpm for 10 min and washing with DI water three times. The precipitates were dialysis for 2 days in order to remove the impurities, and the GS-MGO was achieved by freeze-drying at −70 °C for 24 h.

### 2.3. Characterization

X-ray diffraction (XRD) patterns of the samples were tested at the 2θ value of 3–80° using a Rigaku Ultima III X-ray power diffractometer, and the data were recorded using a Cu Κα X-ray tube with a wavelength of 1.5406 Å, running at 40 KV and 30 mA. Fourier transform infrared (FTIR) of the samples was performed on a Spectrum II FTIR Spectrometer (Perkin Elmer, Waltham, MA, USA) at a resolution of 2 cm^−1^ over the wavenumber range of 4000 cm^−1^–400 cm^−1^. Raman spectra of samples were detected using a DXR Smart Raman spectrometer (Thermo Fisher, Waltham, MA, USA) with laser excitation of 532 nm.

### 2.4. Antibacterial Activity

*Escherichia coli* ATCC 25922 (*E. coli*), *Staphylococcus aureus* ATCC 6538 (*S. aureus*), and *Listeria monocytogenes* ATCC 19115 (*L. monocytogenes*) in Tryptone Soya Broth (TSB, Oxoid, Hampshire, UK) suspensions with a concentration of 1 × 10^5^ CFU/mL were pre-cultures for the antibacterial activity determination.

#### 2.4.1. Disk Diffusion Analysis

A total of 10 mg sterilized GS-MGO was loaded on filter paper discs (diameter of 10 mm). Then, 60 μL of *E. coli*, *S. aureus,* and *L. monocytogenes* suspensions were spread on Tryptone Soya Agar (TSA, Oxoid, Hampshire, UK), respectively. Then, the samples were placed on the TSA plates and incubated at 37 °C for 24 h. Any leaching effect was estimated by observing the inhibition zone.

#### 2.4.2. Antibacterial Growth Effect

GS-MGO with different amounts was added into 40 mL of *E. coli*, *S. aureus*, and *L. monocytogenes* suspensions and incubated at 37 °C for 9 h at 100 rpm. A total of 3 mL supernatants were taken out each hour, and the optical density was measured at a wavelength of 600 nm (OD_600_) using a JINGHUA UV-754 (Shanghai Jinghua Technology Instrument Co., Ltd., Shanghai, China). The absorbance was recorded.

#### 2.4.3. Plate Count Method

GS-MGO were added into 40 mL pre-cultured bacterial suspensions and placed in a 37 °C incubator and shaken at 100 rpm for 8 h. At the same time, the controls were hatched using the same institution with no sample addition. Then, 100 μL diluted bacterial suspensions were spread on the TSA plates and the number of colonies cultured on the plate after 24 h was counted. The inhibition rate (*P*) was calculated by Equation (1):

(1) $$P\;(\%)=\frac{B-C}{B}\;\times \;100\%$$

where *B* is the colony numbers for the control, and *C* is the colony numbers for the experimental group.

### 2.5. Recyclable Activity

The recyclable activity of the bacteriostatic test was carried out continuously eight times by measuring the bacterial optical density (OD_600_). The inhibition effect was compared with the control (without any treatment), and the recyclability was evaluated.

### 2.6. Protein Leakage

The bacterial suspensions with an exponential growth phase of 1 × 10^8^ CFU/mL were centrifuged, and 0.85% NaCl solution was added to the remaining bacteria to achieve an OD value of 1.5 after the supernatant was removed. Then, 25 mg samples were added for 1.5 h incubation, and a control group was set without adding samples. A total of 40 μL of supernatant was added to 400 μL of BCA working solution and allowed to stand at 37 °C for 30 min. The absorbance was read at 562 nm.

## 3. Results and Discussion

### 3.1. Characterization

**Figure 2:**
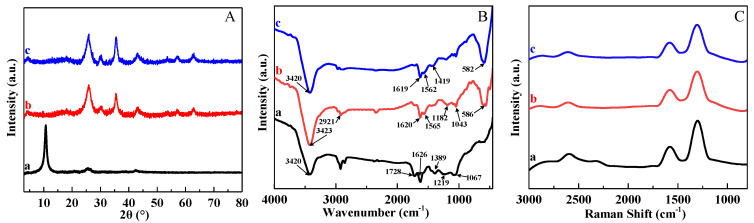
XRD patterns (**A**), FTIR spectra (**B**), and Raman analysis (**C**) of GO (a), MGO (b), and GS−MGO (c) nanohybrids.

The structural information of GO, MGO, and GS-MGO was characterized by XRD. As displayed in Figure 2A, a sharp diffraction peak appeared at 2θ = 10.6° (curve a), corresponding to the (002) crystal reflection of GO [19]. The XRD pattern of MGO (curve b) displayed several diffraction peaks at 2θ = 30.3°, 35.6°, 43.2°, 57.2°, and 62.9° which are assigned to (220), (311), (400), (511), and (440) planes of the cubic lattice of magnetite Fe_3_O_4_ (JCPDS No. 019-0629), respectively [20,21]. The diffraction peak of GO disappeared since the GO was reduced by the ammonia in the system. Thus, the existence of Fe_3_O_4_ NPs in the MGO nanocomposite is confirmed. For GS-MGO, similar characteristic peaks were found in the XRD spectrum; all the characteristic magnetic peaks of GS-MGO (curve c) were clearly observed. Therefore, the high purity of the prepared GS-MGO nanohybrids is verified.

FTIR spectra of GO, MGO, and GS-MGO composites are presented in Figure 2B. For GO (curve a), the peak at 3420 cm^−1^ was clearly observed, which is attributed to the stretching vibration of O-H [22]. The peaks at 1728 cm^−1^, 1626 cm^−1^, and 1389 cm^−1^ correspond to carboxyl C=O, aromatic carbon C=C, and in-plane bending of C-OH, respectively [23,24]. The characteristic peak signals that appeared in 1219 cm^−1^ and 1067 cm^−1^ are assigned to the C-OH and C-O stretching vibrations [25,26]. In the case of MGO (curve b), the characteristic peaks of functional groups were similar to those of GO, except a new peak at 586 cm^−1^ was observed, which was assigned to the Fe-O stretching vibration [27,28]. For GS-MGO (curve c), two bands of 1619 cm^−1^ and 1562 cm^−1^ corresponding to C=O and N-H of amide appeared on its spectrum [29,30]. Thus, GS is proved to be successfully grafted onto MGO.

Raman spectroscopy was used to analyze the structural changes of the compounds [31]. The spectra of GO, MGO, and GS-MGO are shown in Figure 2C. GO (curve a) showed two characteristic peaks at 1342 cm^−1^ and 1588 cm^−1^, corresponding to the D band and G band [4,32], respectively. The D band refers to the disordered structure of graphene due to the sp^3^ hybridization, presenting the existence of structural defects [33]. The G band resulted from the elongation of the bond between the sp^2^ carbon pair in the ring and the chain. Compared to the MGO (curve b), the I_D_/I_G_ decreased obviously, which could be caused by the partial reduction of GO by ammonia during the preparation process. It was also observed that MGO grafted with GS (curve c) had similar D and G characteristic bands as MGO. It is possible because the GS grafting occurred in the amidation reaction between the amino group of GS and the carboxyl group of MGO, leading the carbon skeleton of GO to be largely retained.

**Figure 3:**
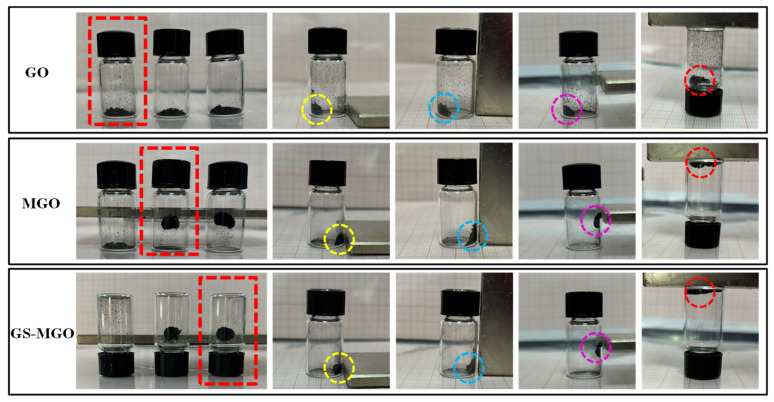
Magnetic performance of GO, MGO, and GS-MGO.

The magnetic properties of GO, MGO, and GS-MGO were verified by indirect contact with external magnets. As shown in Figure 3, GO was placed on the left side of the reagent bottle. GO stood still without any movement to approach the magnet when the magnet approached from the right side and changed its horizontal or vertical mode. When the magnet was placed in the middle of the right side, GO was not attracted to the magnet. Then, the reagent bottle was placed upside down, and the magnet was put on top. GO slipped from the top and was not attracted by the head magnet, indicating that GO was not magnetic. For MGO, its magnetism was tested in the same way. As the magnet approached, MGO quickly migrated from the left to the right and adhered to the inside of the reagent bottle. When the magnet on the right side was suspended or placed on the top, MGO always maintained the behavior of attracting to the magnet, indicating that Fe_3_O_4_ had been successfully modified onto GO and the prepared MGO exhibited good magnetic properties. Moreover, the GS-MGO hybrids also had the same magnetic action as MGO, indicating that the magnetism was maintained during the amidation reaction between GS and MGO.

### 3.2. Antibacterial Performance

**Figure 4:**
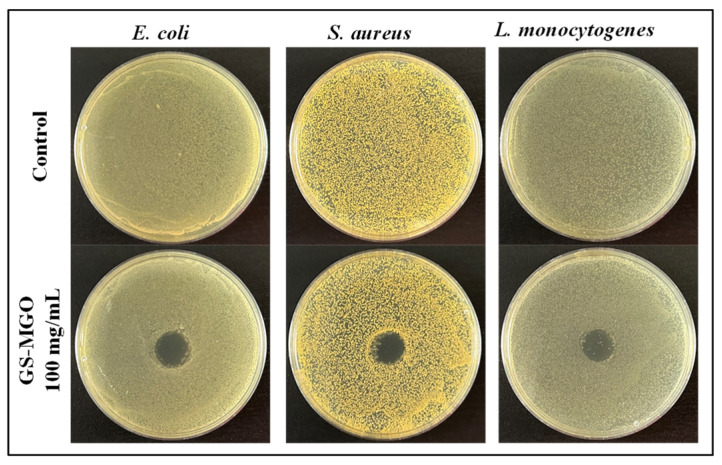
The inhibition zones of GS-MGO against *E. coli*, *S. aureus*, and *L. monocytogenes*.

The disk diffusion method was first used to evaluate any leaching effect of the prepared GS-MGO, and the result is shown in Figure 4. After 24 h of incubation, no observed inhibition zones were found on the TSA plates cultured with *E. coli*, *S. aureus,* and *L. monocytogenes*. This indicates that the prepared GS-MGO expressed a non-leaching effect of GS.

**Figure 5:**
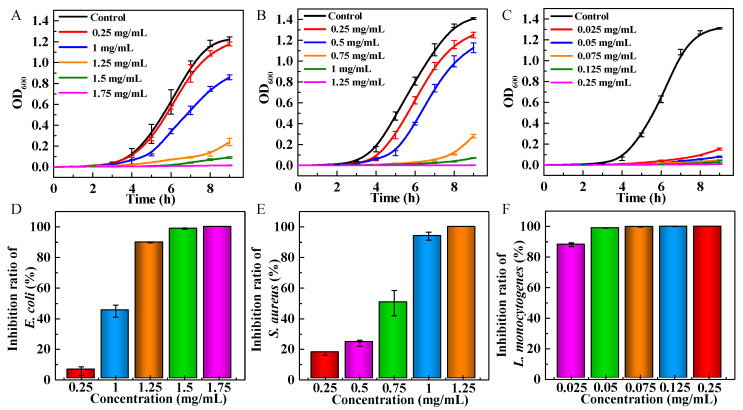
Bacterial growth curves and inhibition ratios of GS-MGO nanohybrids against *E. coli* (**A**,**D**), *S. aureus* (**B**,**E**)*,* and *L. monocytogenes* (**C**,**F**) with different concentrations.

The bacterial growth curve can intuitively express the inhibitory effect of the antibacterial agent on the bacteria in the lag phase, log phase, and stationary phase. The standard growth curves with three stages are seen in Figure 5. With the increase in GS-MGO concentration, the inhibitory effect was more and more obvious in *E. coli*, *S. aureus,* and *L. monocytogenes*. For *E. coli* (Figure 5A), there was a significant lag phase and exponential phase inhibition when the addition concentration was 1.25 mg/mL. When the concentration of GS-MGO reached 1.75 mg/mL, the complete inhibition effect appeared. Similarly, the inhibitory effect of two periods is observed in Figure 5B, showing that prolonged lag and log phases were displayed in the bacterial growth curve of *S. aureus* when the concentration of GS-MGO was 0.75 mg/mL, and the complete inhibition of *S. aureus* was displayed with the concentration of 1.25 mg/mL. Interestingly, the inhibitory effect on *L. monocytogenes* of GS-MGO was the most obvious in that the complete inhibition of *L. monocytogenes* was achieved when the concentration was 0.125 mg/mL (Figure 5C). The successful inhibition of these three bacteria verified that GS-MGO was an excellent antibacterial agent that could not only achieve an excellent antibacterial effect on Gram-negative bacteria but also expresses strong inhibition ability on Gram-positive bacteria, especially *L. monocytogenes*. In fact, when the concentration of MGO was 0.25 mg/mL, the inhibition ratios of *E. coli*, *S. aureus*, and *L. monocytogenes* were 5.7%, 17.3%, and 100%, respectively. Obviously, it had a stronger inhibitory effect on Gram-positive bacteria than on Gram-negative bacteria. This phenomenon could probably be due to the structural differences in the outer membrane of bacteria. Gram-negative bacteria have an external lipid membrane which acts as an extra barrier to the antibacterial effects of the GS-MGO, while Gram-positive bacteria have a thick peptidoglycan layer without any external lipid membrane leading to greater sensitivity.

**Figure 6:**
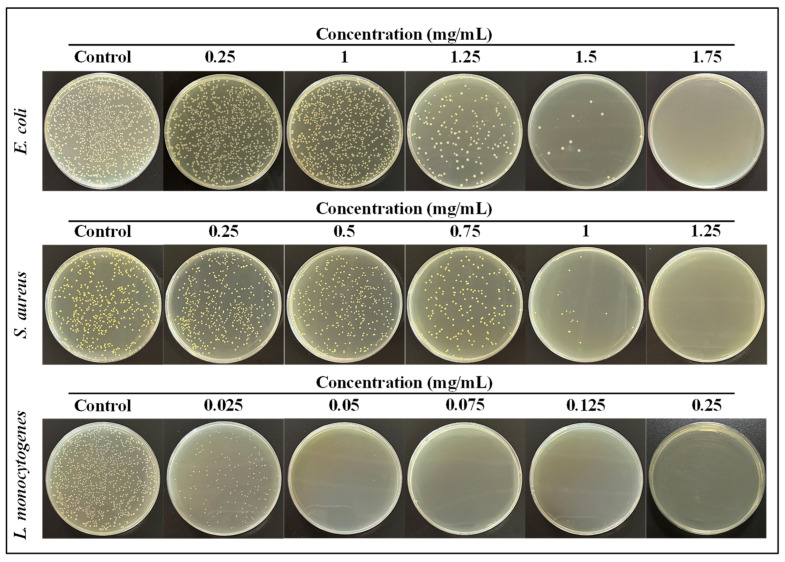
The bacterial colonies of GS-MGO nanohybrids against *E. coli*, *S. aureus*, and *L. monocytogenes* with different concentrations.

The plate counting method was applied to determine the inhibition ratios of the GS-MGO nanohybrids on *E. coli*, *S. aureus,* and *L. monocytogenes*. As shown in Figure 6, the 10^−5^ dilutions of the tested bacteria after the treatment of GS-MGO nanohybrids with different concentrations were spread on the TSA plates, and the bacterial colonies were clearly observed after 24 h incubation. With the increase in GS-MGO concentration, the number of colonies decreased. For *E. coli*, when the addition concentration of GS-MGO was 1.25 mg/mL, there was a significant reduction in the number of colonies, and the calculated bacteriostatic ratio achieved 89.8% (Figure 5D). When the addition concentration reached 1.5 mg/mL, the antibacterial ratio reached 98.8%. Similarly, when the addition concentration of GS-MGO was 1.75 mg/mL, no colony was observed at all. The inhibition ratio at this time was 100%, indicating that GS-MGO achieved the complete inhibition of *E. coli*, which is consistent with the result in Figure 5A. For *S. aureus*, when the addition concentration of GS-MGO was 0.75 mg/mL, the inhibition ratio was 50.2%, as shown in Figure 5E. When the addition concentrations of GS-MGO were 1 mg/mL and 1.25 mg/mL, the inhibition ratios reached as high as 93.9% and 100%, respectively. The corresponding bacterial colonies are displayed in Figure 6. Surprisingly, GS-MGO exhibited a distinguished antibacterial effect against *L. monocytogenes* (Figure 5F). When the concentration of GS-MGO was only 0.025 mg/mL, the inhibition ratio was as high as 88.1%. When the added concentration of GS-MGO increased to 0.05 mg/mL, 99% of the antibacterial activity was expressed (Figure 5F), which is in agreement with the different sensitivities of the prepared GS-MGO nanohybrids towards Gram-positive bacteria and Gram-negative bacteria.

### 3.3. Recyclable Activity

**Figure 7:**
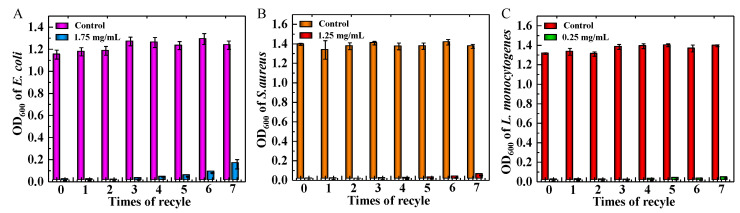
Recyclable antibacterial activity of GS-MGO against *E. coli* (**A**), *S. aureus* (**B**), and *L. monocytogenes* (**C**) with different concentrations.

Recyclability is one of the most important factors for antibacterial agents, which can characterize the upper limit of continuous use of GS-MGO. Based on the above studies, the concentrations of GS-MGO chosen for the recyclable antibacterial activity against *E. coli*, *S. aureus,* and *L. monocytogenes* were different but optimal with excellent antibacterial properties. The eight times of GS-MGO antibacterial activities were determined by measuring OD_600_ after 9 h incubation, and the results can be clearly observed in Figure 7. It was not difficult to find that the antibacterial activity of the prepared GS-MGO slightly decreased with the number of cycles, especially for *E. coli*, but they still had a high level of antibacterial ability even after eight times applications. The slight decrease might be due to the inevitable loss of GS-MGO nanohybrids in every cycle. However, the high recyclability of GS-MGO and its strong inhibition effect against Gram-negative and Gram-positive bacteria exhibited great potential in the field of new non-leaching antimicrobial agents. In particular, the expression of a more sensitive and efficient killing level for *L. monocytogenes* can effectively expand the practicality of the microbial food packaging field.

### 3.4. Protein Leakage Analysis

When bacteria die, the complete structure is destroyed so that the internal contents, such as proteins and nucleic acids, leak into the external environment. The leaked proteins could be detected with a BCA kit. Cu^2+^ can be reduced by proteins to form Cu^+^, forming a purple complex with BCA under alkaline conditions, and its absorbance can be measured at 562 nm. The antibacterial effects of GS-MGO nanohybrids on *E. coli*, *S. aureus*, and *L. monocytogenes* were further studied by determining the leaked proteins.

**Figure 8:**
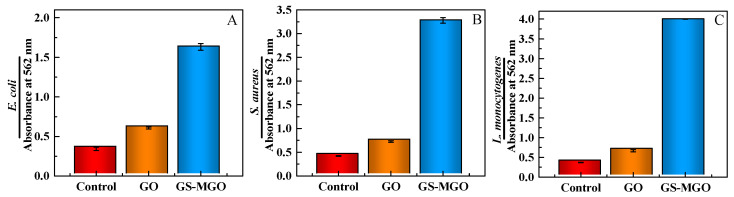
The absorbance values of leaked proteins treated with the control, GO, and GS-MGO nanohybrids against *E. coli* (**A**), *S. aureus* (**B**) and *L. monocytogenes* (**C**).

The protein leakage experiment was carried out, and the results are shown in Figure 8. The UV absorption value of *E. coli* in the control group was 0.344, indicating that the protein leakage is very small with good bacterial cell activity. After adding GS-MGO, the UV absorption value increased to 1.632 due to a large amount of protein leakage, which indicates that the cell membranes of *E. coli* were seriously damaged and a large number of bacteria died. Similarly, for *S. aureus*, the absorption value of the control group was 0.419, and the absorption value after GS-MGO treatment was 3.28. At this time, the cell membranes were almost destroyed, resulting in a large amount of protein leakage, which eventually caused the death of the bacteria. Moreover, GS-MGO had the best antibacterial property against *L. monocytogenes*, and the UV absorption value after the treatment reached as high as 3.998, which is identical to the above antibacterial results.

## 4. Conclusions

In summary, magnetic GS-MGO antibacterial agents were prepared with GS grafting to Fe_3_O_4_ NPs loaded GO. The prepared GS-MGO nanohybrids exhibited excellent inhibition ability against Gram-negative bacteria *E. coli* and Gram-positive bacteria *S. aureus* and *L. monocytogenes* by destroying bacterial cell membranes. In particular, it showed more sensitive antibacterial activity against *L. monocytogenes*. The recycling antibacterial ability of GS-MGO nanohybrids was confirmed with good non-leaching performance. Therefore, the prepared GS-MGO nanohybrids could be used as an excellent antibacterial agent. They displayed great potential in clinical environmental applications and food packaging fields to inhibit bacterial growth and propagation effectively without any leaching effect. This study provides an effective strategy for the design and development of a novel non-leaching antibacterial agent.

## Data Availability

Data will be available upon valid request.
